# A Guide for Using Flight Simulators to Study the Sensory Basis of Long-Distance Migration in Insects

**DOI:** 10.3389/fnbeh.2021.678936

**Published:** 2021-06-10

**Authors:** David Dreyer, Barrie Frost, Henrik Mouritsen, Adrien Lefèvre, Myles Menz, Eric Warrant

**Affiliations:** ^1^Lund Vision Group, Department of Biology, University of Lund, Lund, Sweden; ^2^Department of Psychology, Queens University, Kingston, ON, Canada; ^3^Institute for Biology and Environmental Sciences, University of Oldenburg, Oldenburg, Germany; ^4^Department of Migration, Max Planck Institute of Animal Behavior, Radolfzell, Germany; ^5^Department of Biology, University of Konstanz, Konstanz, Germany; ^6^School of Biological Sciences, The University of Western Australia, Crawley, WA, Australia; ^7^Research School of Biology, Australian National University, Canberra, ACT, Australia; ^8^Division of Information, Technology and Development, University of South Australia, Adelaide, SA, Australia

**Keywords:** orientation, navigation, insects, sensory ecology, behavior

## Abstract

Studying the routes flown by long-distance migratory insects comes with the obvious challenge that the animal’s body size and weight is comparably low. This makes it difficult to attach relatively heavy transmitters to these insects in order to monitor their migratory routes (as has been done for instance in several species of migratory birds. However, the rather delicate anatomy of insects can be advantageous for testing their capacity to orient with respect to putative compass cues during indoor experiments under controlled conditions. Almost 20 years ago, Barrie Frost and Henrik Mouritsen developed a flight simulator which enabled them to monitor the heading directions of tethered migratory Monarch butterflies, both indoors and outdoors. The design described in the original paper has been used in many follow-up studies to describe the orientation capacities of mainly diurnal lepidopteran species. Here we present a modification of this flight simulator design that enables studies of nocturnal long-distance migration in moths while allowing controlled magnetic, visual and mechanosensory stimulation. This modified flight simulator has so far been successfully used to study the sensory basis of migration in two European and one Australian migratory noctuid species.

## Introduction

Like the North American Monarch butterfly, many species of moths have been identified as long-distance migrants (Williams, 1958). Naturalistic observations, and comprehensive recordings of flight trajectories using vertical-looking radar, have demonstrated the migratory directions of insects are not necessarily determined by the prevailing wind direction (Chapman et al.,2008a,b, 2010). In fact many insects have some level of control over their desired migratory route, an ability that implies the use of a compass that enables individuals to steer a course during a migratory flight (Chapman et al.,2008a,b, 2015). While the compass systems of some diurnal migratory Lepidopterans, such as the Monarch butterfly (*Danaus plexippus*) or the Painted Lady (*Vanessa cardui*), are relatively well described (e.g., Mouritsen and Frost, 2002; Reppert et al., 2004; Stalleicken et al., 2005; Nesbit et al., 2009; Mouritsen et al., 2013), little is known about the compass cues and the navigational mechanisms that enable the migrations of nocturnal migrants such as moths.

One such nocturnal migrant is the Australian Bogong moth (*Agrotis infusa*), a remarkable nocturnal navigator (see portrait in Figure 6A). After emerging from its pupa in early Spring, somewhere within the semi-arid breeding grounds of inland south-eastern Australia, an adult Bogong moth embarks on a long migration toward the Australian Alps (Common, 1954; Warrant et al., 2016). Because the breeding grounds of Bogong moths are so vast, this journey will occur in one of many possible directions, anywhere between the extremes of directly east (from western Victoria) to southwest (from southeast Queensland), depending on where the journey begins. Migratory flights may take many nights or even weeks and cover over 1000 km. Once the Bogong moths have arrived in the Alps (starting in early October), they seek out the shelter of high ridge-top caves and rock crevices (typically at elevations exceeding 1800 m). In their hundreds of thousands, moths line the interior walls of each alpine cave where they aestivate over the summer months, probably to escape the heat of the Australian plains (Tomlinson et al., in preparation). Toward the end of the summer (February and March), the same individuals which arrived months earlier emerge from the caves and begin their long return trip to their breeding grounds. Once arrived, the moths mate, lay their eggs, and die. The next generation of Bogong moths – hatching in the following Spring – then repeat the migratory cycle afresh. Despite having had no previous experience of the migratory route, these moths find their way to the Australian Alps and locate the aestivation caves dotted along the high alpine ridges of south-eastern Australia.

To navigate to a specific alpine destination, through unknown territories or environments, Bogong moths need to rely on external compass cues (Warrant et al., 2016; Dreyer et al., 2018b). To study these cues, we modified a previously invented system, the Mouritsen-Frost flight simulator (Mouritsen and Frost, 2002; Minter et al., 2018). The original Mouritsen-Frost flight simulator consists of a cylindrical behavioral arena (placed on an experimental table) which is equipped with a vertical axle to which a flying moth is tethered, and an optical encoder. The encoder is connected to the top of the axle, which continuously measures the flight direction of the moth relative to geographic or magnetic North, thus allowing the reconstruction of the moth’s virtual flight path. The modified Mouritsen-Frost flight simulators we describe here added a projector system, a clear Plexiglass tabletop, a mirror and control software which enables the experimenters to simulate the optic flow of the landscape beneath the moths. This optic flow continuously adjusts its direction to match the direction the moth is heading at any moment in time. The flight simulator’s simple and compact design not only allows deployment in the field, but also in the lab where it can be incorporated within more sophisticated assemblies where stimulation can be controlled, such as within a magnetic coil system, or even incorporated with an electrophysiology rig (Beetz et al. in preparation).

In this paper we describe in detail how a modified Mouritsen-Frost flight simulator is built, the various experiments it can be used for and the types of data it can produce (and how these data can be analyzed). This description will be largely based around our ongoing work on the Australian Bogong moth, and various European relatives, but the equipment and analyses are applicable to a wide variety of flying insects.

### The Modified Mouritsen-Frost Flight Simulator

Since one of our main experimental goals was to investigate the magnetic sense of night-flying insects, the entire setup was built from non-magnetic materials.

### The Behavioral Arena

**FIGURE 1:**
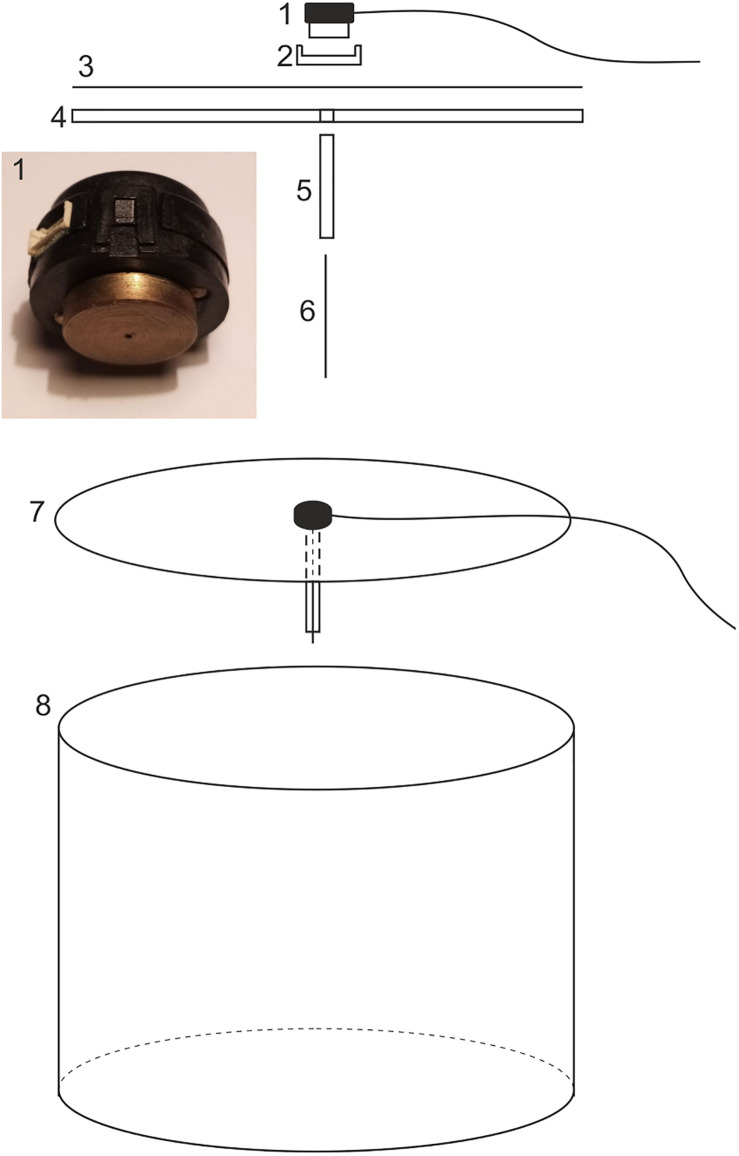
A schematic drawing of the flight simulator showing the encoder (1), the encoder mount (2), the diffuser paper (3), the circular Plexiglass lid (4 and 7), the protective brass shaft (5), the tungsten axle (6), and the behavioral arena (8). For explanation see text.

**FIGURE 2:**
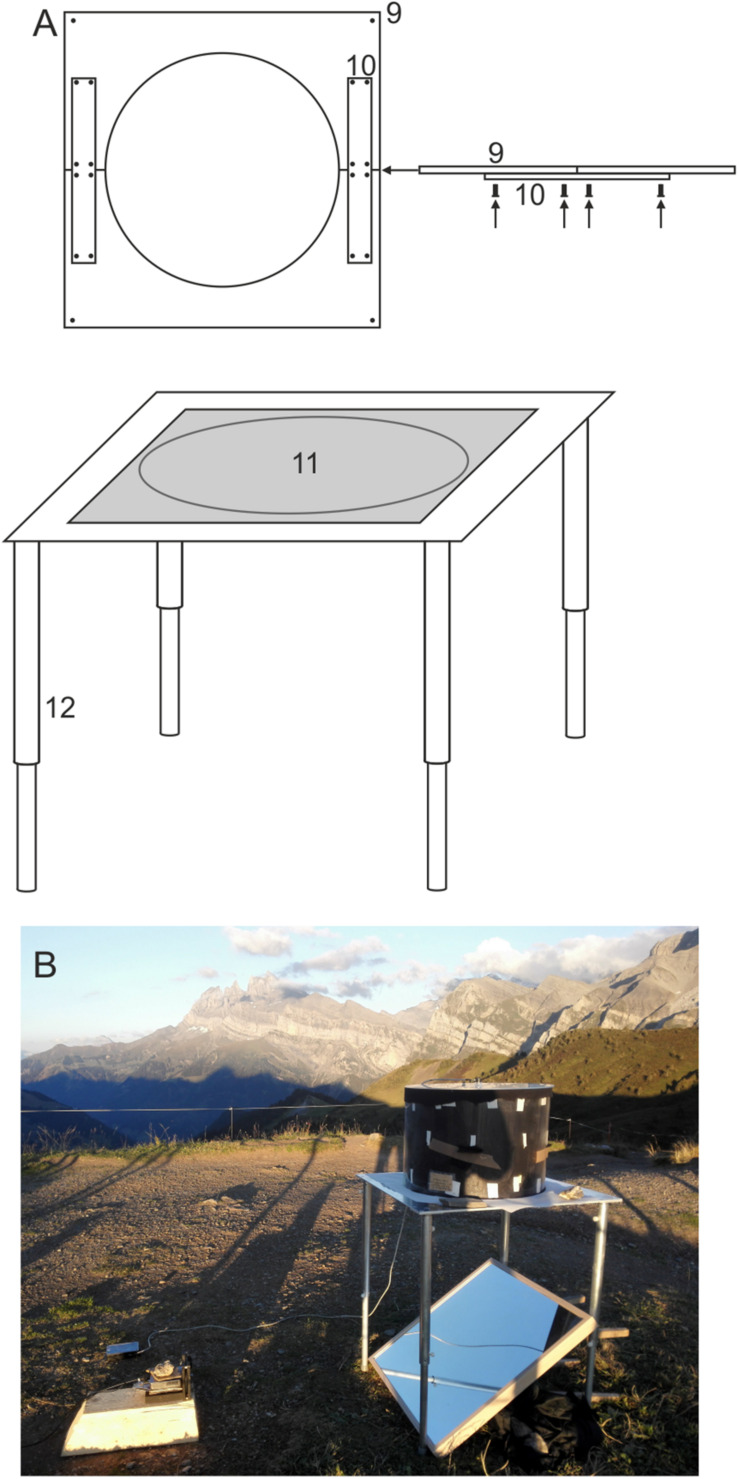
The experimental table. **(A)** Schematic drawing of the experimental table showing the tabletop (9), the aluminum connectors (10), the circular opening (11), and the telescopic legs (12). **(B)** A photograph showing its deployment in the field at Col de Coux, Switzerland. For detailed explanation see text.

A length of wide Plexiglass cylinder (or any other type of plastic cylinder) can be used as an arena. The dimensions of this cylindrical arena are more or less arbitrary, but we have achieved good results using a cylindrical Plexiglass arena of diameter 500 mm and height 360 mm (*8* in Figure 1; 5 mm material thickness) placed vertically on an experimental table (Figure 2). The interior design of the arena is of particular importance since moths are extremely sensitive to visual landmarks and will steer their course relative to any larger visible landmark on the inside wall of the arena. We thus avoided having a glossy interior wall (to reduce reflections) or a wall covered in paper or cardboard which can buckle. In order to minimize landmarks, we covered the interior wall of the arena with a uniform self-adhesive black felt, where the visibility of the join was minimized.

### The Encoder Mount

**FIGURE 3:**
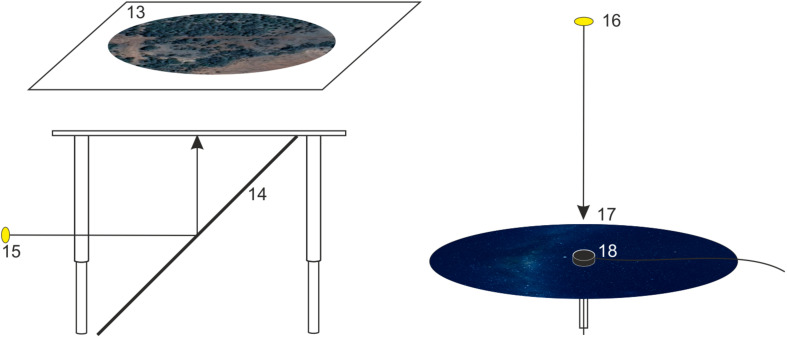
A schematic drawing showing how optic flow (left) and an austral starry night sky (right) are projected onto the experimental arena. Moving optic flow (a satellite image of the Australian countryside) is projected from a projector placed to the side of the table (15), via a 45° mirror (14), onto the underside of a diffusing screen (13) placed on the tabletop under the behavioral arena. A local starry night sky (generated using the planetarium software Stellarium) is projected from a projector mounted above the arena (16) onto a circular diffusing screen (17) placed on top of the arena (which also holds the encoder mount (18) at its center).

The optical encoder (described in detail below) is held within an encoder mount at the center of the upper opening of the cylindrical arena. The encoder-mount design is of equal importance as the design of the inside wall of the arena since this mount constitutes a very dominant landmark if a non-symmetrical design is chosen. In earlier experiments, we used a simple transparent Plexiglas beam as an encoder mount, which was placed across the diameter of the open arena top. Unfortunately this introduced a bipolar landmark. The easiest way to avoid this is to place a circular lid on the arena with the encoder mounted at its center. We used a circular sheet of UV-transparent Plexiglass (*4* and *7* in Figure 1 and *17* in Figure 3; 510 diameter × 4 mm thick) as the lid (and encoder mount). Topped with Lee filter diffuser paper (*3* in Figure 1), this mount can also serve as a projection screen if dorsal visual stimulation is desired (see below). In our setup, the cylindrical casing of the encoder is held in place at the center of the lid by a custom-machined plastic cylindrical mount equipped with a grub screw to fix the encoder (*2* in Figure 1). A hole drilled through the center of the lid allows a 110-120 mm long brass tube (5 mm outer diameter – *5* in Figure 1) to be inserted through this hole, and fixed to the Plexiglass sheet with super glue. This thin cylindrical tube surrounds and protects a long (130 mm) tungsten rod (*6* in Figure 1) connected to the rotational axis of the optical encoder (*1* in Figure 1). The tungsten rod serves as the axle of the optical encoder and is attached to the dorsal thoracic surface of the moth (see below for details).

### The Experimental Table

The design of the table (Figure 2) is more or less arbitrary as well, as long as it features a circular opening at the center of the tabletop that has the same diameter as the circular arena and has sufficient clearance underneath to position a suitable mirror (see Figure 3). After testing many different table designs, we settled on using custom-machined lightweight aluminum tables (700 **×** 700 **×** 4 mm aluminum tabletop featuring a 490 mm circular opening at the center) with telescopic legs (850 mm length, if fully elongated) made out of two aluminum pipes (*12* in Figure 2; pipe 1: 4 cm outer diameter, 50 cm length; pipe 2: 45 cm length) for maximum flexibility. The choice of aluminum has the added advantage that it is non-magnetic and thus suitable for experiments involving magnetic stimulation. The telescopic legs were useful for leveling the table on uneven ground during outdoor field experiments. The tabletop (*9* in Figure 2) was cut into two halves for easy transport (35 **×** 70 cm each) - it can be easily re-assembled using aluminum connectors (*10* in Figure 2). The legs can be disassembled from the tabletop and reconnected using screws. This table can easily be transported in a large suitcase.

### Projecting Optic Flow and the Starry Night Sky

In our experiments, we have been interested in the use of stars as compass cues during the long-distance migration of Bogong moths. To create overhead starry night-sky stimuli we use a portable ASUS S1 LED projector situated 1.3 m above the arena (located at *16* in Figure 3) and connected to a laptop via a HDMI cable (3–5 m). To block any stray light from the projector itself, the projector is enclosed within a 3D-printed plastic box with air vents to allow cooling and featuring an opening in front of the lens. This combination of box and projector can be mounted on an adjustable tripod or a ball joint mount (available from Thorlabs) using the typical 1/4” screw for camera/projector mounts.

To simulate the starry sky over our experimental site on the date and time of our experiments, we used the freeware planetarium software *Stellarium* and created screenshots (screen resolution 7480 **×** 720 pixels) of these simulated starry skies. These were then cut into a circular shape using Corel Draw X5 and saved as PNG files (300 dpi) to create the stimulus images. These circular images were then projected onto a screen placed on top of the arena. This screen consists of a circular lid of clear UV-transmissive Plexiglass topped with UV-transmissive diffusing paper (Lee Filters 250 half-white diffuser) having a diameter of 50 cm (*17* in Figure 3). Since the projector does not emit UV light, and we wished to have the full spectrum of light available from the night sky available within our stimulus, we installed a custom-made LED-ring (built by Timothy McIntyre, University of South Australia: outer diameter 120 mm, inner diameter 50 mm) featuring eight UV LEDs (LED370E Ultra Bright Deep Violet LED; Thorlabs) centered over the exit opening of the 3D-printed plastic box containing the projector. The brightness of the LED-ring was controlled using custom software written in MATLAB (Mathworks, Natick, MA, United States) together with several layers of neutral density filters (Lee Filters) which were fixed to the front of the LED-ring (thus allowing the intensity of UV illumination to be adjusted to natural nocturnal levels).

We have found that the presence of dim, slowly moving optic flow, projected beneath the moth and always moving from nose to tail irrespective of the moth’s orientation in the arena, provides extra motivation for the moths to fly (see below). A second ASUS S1 LED projector (also encased within a 3D-printed plastic box and located at *15* in Figure 3) projects ventral optic flow via a 45° mirror. This mirror (*14* in Figure 3; IKEA model NISSEDAL, 65 **×** 65 cm) deflects the projection of the optic flow onto a screen situated underneath the arena. This screen consists of a transparent Plexiglas plate (*11* in Figure 2; 60 **×** 60 **×** 0.5 cm) covered with one layer of white opaque diffuser paper (Lee Filters 250 half-white diffuser). The intensity of the optic flow is dimmed to nocturnal levels by using a combination of several neutral density filters (Lee Filters) placed over the exit opening of the 3D-printed plastic box containing the projector.

### The Recording System

**FIGURE 4:**
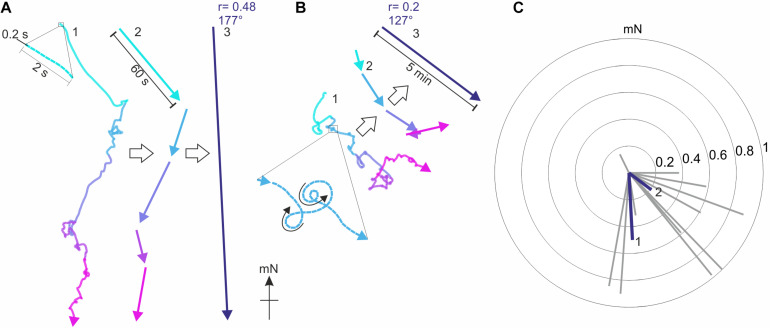
Typical virtual flight tracks recorded by the encoder system. **(A)** The virtual flight track of a Red underwing moth (*Catocala nupta*, RU#11) recorded in Illmitz (Austria) over 5 min of consecutive flight (each minute is represented by a different *color*), plotted relative to magnetic North (mN). In 1, the entire 5 min flight track is shown with the moth’s flight direction recorded every 0.2 s (see *enlargement*), while in 2 the resultant vectors calculated for each minute of the same track are shown. 3 shows the resultant flight trajectory vector of RU#11 (*r* = 0.48, α = 177°), based on the 0.2 s samples recorded over 5 min of consecutive flight. **(B)** As in A, but for the track of another Red underwing moth (RU#5) recorded at the same location. This particular individual was less oriented than RU#11, as seen in the comparably shorter lengths (i.e., lower *r* values) of the resultant vectors in 2 and 3. Note that even though moth RU#5 flew in many loops (see *enlargement* in 1), it was able to fly both clockwise and counter-clockwise (*black arrows* in 1), a good indicator that the stalk was attached symmetrically to the thorax of the moth and that neither of the wings were damaged. **(C)** The vectors of 14 Red underwings are plotted as *gray radial lines* in a circular diagram [the vectors of RU#11 (1) and RU#5 (2) are plotted in *blue*]. The radii of the *concentric circles* indicate the *r* value (from 0 to 1) at increasing step-size from the center toward the periphery. Based on these 14 vectors, we can also investigate the orientation behavior of the moths as a single population by employing the Moore’s modified Rayleigh test (see Figures 7, 8), which accounts not only for the direction of each moth (as in a classical Rayleigh test) but also for its directedness (i.e., its flight vector *r* value).

Our recording system is based on optical encoder systems from US Digital. Our preferred system is their E4T Miniature Optical Kit Encoder (located at *18* in Figure 3) in combination with their USB4 Encoder Data Acquisition USB Device, including all necessary cables. The standard encoder software US Digital Explorer shows the orientation of the encoder axle (or moth) as a compass needle that rotates relative to North within a circular compass rose. In order to fix the tungsten encoder axle (*6* in Figure 1) to the encoder and have it rotate freely without jamming, a cylindrical piece of brass (14 mm diameter, 4 mm height), equipped with a tiny hole (1 mm diameter) for the tungsten axle, was glued to the underside of the encoder. The encoder has an angular resolution of 3°, so the output values of the system (2 channel quadrature TTL square-wave outputs which are converted into degrees by the software) range between 0 and 120 rather than 0° to 360°. This means that each output value in degrees has to be multiplied by 3 in the analysis to fit the data into a full circle reference frame. During our experiments, several Microsoft operating systems (Windows XP, Windows 7 and Windows 10) have been used as a platform for the recording software. Since some of our experiments take place in the field, we use a “semi-rugged” laptop model (Dell Latitude E6430 ATG) for our recordings. The output file format is a standard text file (.txt) in which the observed heading directions are saved in a column together with a complementary timestamp. We measure the heading directions at a sampling rate of 5 Hz. Thus, over a period of typically 5 to 10 min, we are able to continuously record a tethered moth’s “virtual flight path,” that is, its heading direction relative to (say) north monitored 5 times per second. From this virtual flight path we are able to construct an average vector representing the moth’s trajectory (Figure 4), the direction and length of which, respectively, reveal the mean orientation angle and directedness of the moth. The directedness of the moth (i.e., its tendency to fly in the same direction) is captured in the *r* value of its trajectory vector, a unitless value between 0 and 1. More directed moths have longer vectors and larger *r* values (e.g., Figure 4A, compared to the less directed moth shown in Figure 4B). How the trajectory vectors of tested moths are used to understand their collective migratory flight behavior will be explained in more detail later.

As mentioned above, we project dim optic flow below the moth (*13* in Figure 3) to simulate an apparent forward movement similar to what a flying insect would experience in the wild, thus promoting flight behavior. The encoder system, while recording the virtual flight paths of the tethered moths, is coupled to the ventral optic flow via a feedback loop. This feedback is maintained by the software package “Flying” (custom written software) that instantaneously adjusts optic flow direction in response to changes in heading direction, thus ensuring that the optic flow always moves backward beneath the tethered moth (head to abdomen) as the moth apparently moves forward. The speed of the optic flow can be adjusted in the “Flying” software, and its illumination intensity (as described above) by neutral density filters. The image we used to create the optic flow was a screenshot taken from Google Earth (set to satellite view; see *13* in Figure 3) – the Earths’ surface near the town of Narrabri (New South Wales, Australia) from an altitude of about 800 m. This town lies close to one of the migratory routes of the Bogong moth.

### Magnetic Stimulation

**FIGURE 5:**
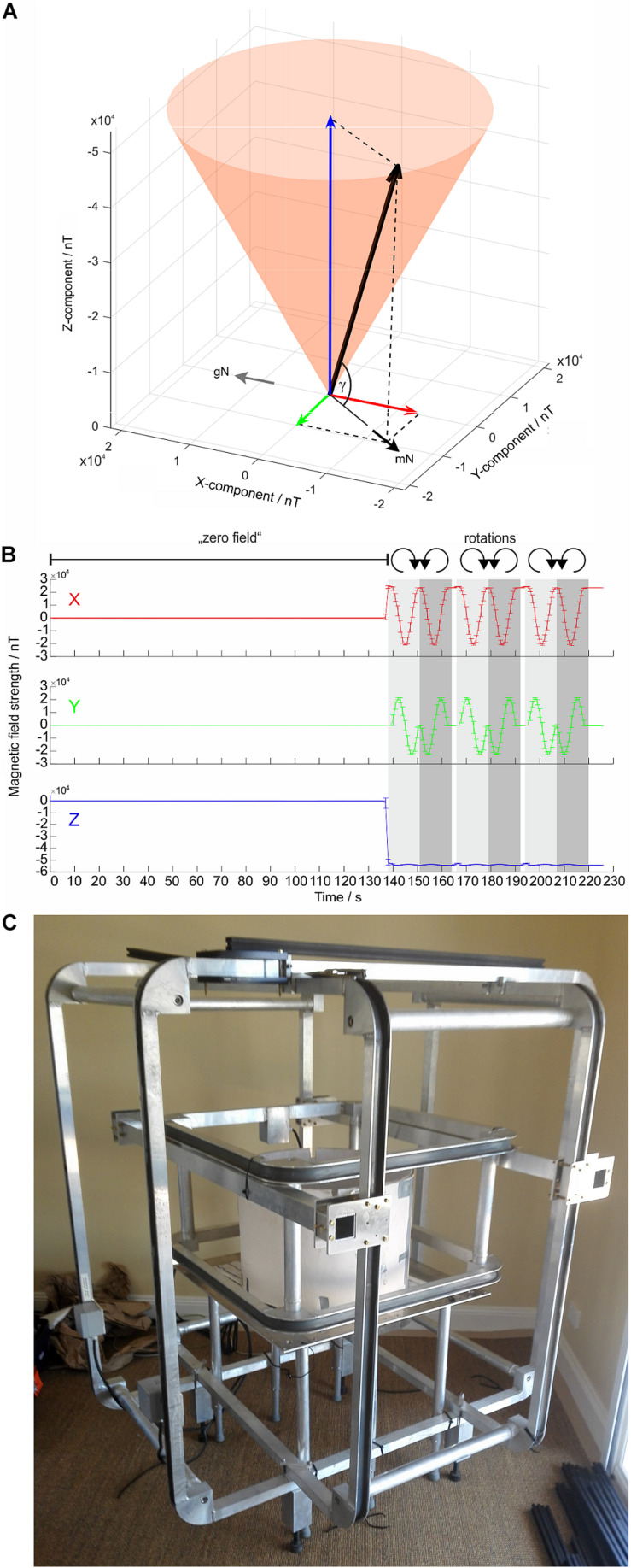
Magnetic stimuli generated by the Helmholtz coil system. **(A)** The experimental magnetic field vector (*thick black arrow*) can be subdivided into 3 vectors (or component vectors) which are oriented perpendicular to each other: the X- (*red arrow*), Y- (*green arrow*), and Z-component (*blue arrow*). The *orange cone* indicates the rotational movement pattern of the resulting magnetic field vector, which points toward magnetic North (mN). **(B)** The magnitude of the X-component (*red arrow*), Y-component (*green arrow*), and Z-component (*blue arrow*) of the experimental magnetic field vector, measured at the center of our Helmholtz coil system, plotted as a function of time for a specific magnetic stimulus sequence (shown here as an example). For the first 2 min of this stimulus sequence, the field was nulled to create a “magnetic vacuum” (zero field). Following the 2-min magnetic vacuum, the Helmholtz coil system was set to generate 3 clockwise (*light gray*) and 3 counter-clockwise (*dark gray*) 360° rotations (12 s each; resolution of magnetic field changes: 1 step per 1°) while keeping inclination γ constant (as in **A**). The *error bars* give the SD around the means of 5 repetitions of the stimulus. Note that the Z-component (and thereby γ) have negative values, reflecting the fact that in the southern hemisphere the field lines of the Earth’s magnetic field exit the Earth’s surface (i.e., inclination angle is defined as being negative). **(C)** A Helmholtz coil system currently in use in Australia with an arena positioned at its center.

**FIGURE 6:**
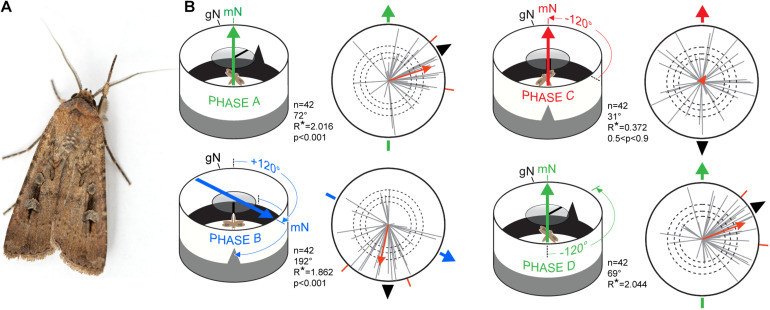
Migratory orientation in Bogong moths is multimodal. **(A)** A male Bogong moth (*Agrotis infusa*). **(B)** Experimental procedure and results. Each tethered moth was subjected to magnetic and visual cues during four 5-min phases (termed phases A–D) and their directions and directedness (orientation and length, respectively, of *gray vectors* in circular plots) measured. When the positions of the magnetic field (*heavy colored arrows*) and visual landmarks (*black triangular ‘mountain’* and *overhead stripe*) are correlated and turned together (Phases A,B,D), the moths (*n* = 42, *gray vectors*) remain significantly oriented near the landmarks (as indicated by the long (highly directed) *red population mean vectors*; *p* < 0.001). When the two cues are set in conflict (Phase C), moths become disoriented (as indicated by the short (undirected) *red population mean vector*; 0.5 < *p* < 0.9). The directedness (length) of the population mean vector is given by its R* value: the greater the R* value, the more directed the population of moths it represents. The R* value also reveals the likelihood that the mean flight direction of a population of moths – where each moth has its own direction and directedness (direction and length of *gray vectors*) – differs significantly from a random, undirected population (according to the Moore’s modified Rayleigh test: Moore, 1980). *Dashed circles*: required α-level for statistical significance (i.e., the R* value required to reliably distinguish the directedness of the population from a random, undirected population): *p* < 0.05, *p* < 0.01, and *p* < 0.001, respectively, for increasing radius. Outer radius of plots: R* = 2.5. *Red radial dashes*: 95% confidence interval. gN, geographic North. mN, magnetic North. Data are from Dreyer et al., 2018b and diagram from Johnsen et al., 2020. The photo of the Bogong moth in A is courtesy of Dr. Ajay Narendra, Macquarie University, Australia.

To test the effects of an Earth-strength magnetic field on the flight behavior of moths, the behavioral arena can be placed within a double-wrapped (Kirschvink, 1992; Mouritsen, 1998; Schwarze et al., 2016), computerized 3D-Helmholtz coil system consisting of three pairs of orthogonally mounted coils: the X-, Y-, and Z-coils (Figure 5C). This computer-controlled Helmholtz coil system enables us to send minute currents through the paired X-, Y-, and Z- coils which result in changes in the magnitude of the respective component vectors (measured in nano Tesla, nT) and thus in changes in the resulting magnetic field vector. By systematically changing the magnitude of the X and Y components (while the Z-component is kept constant), the orientation of the experimental magnetic field vector can be rotated around the Z-axis (clockwise or counter-clockwise), executing a motion pattern which is depicted as a shaded orange cone in Figure 5A. The horizontal orientation of the experimental magnetic field vector (which we define as pointing to magnetic North, mN) can therefore be set to any desired azimuth relative to geographic North (gN in Figure 5A) without altering the total intensity (the magnitude) of the experimental magnetic field vector or the inclination angle (γ in Figure 5A), both of which are maintained at natural local values. Other stimulus designs are also possible – one could for instance include a change of γ without altering the azimuth of the experimental magnetic field vector. In addition to accurately producing and adjusting natural geomagnetic fields within the flight arena, the coils are also able to create a “magnetic vacuum” (i.e., a nulled, or zeroed field; Mouritsen, 1998) around the moth (see Figure 5B). This stimulus (or rather, lack of stimulus) is useful for disabling the magnetic sense if one wishes to test the responses of moths to other relevant compass cues in isolation, such as visual cues or wind. Moreover, our previous work (Dreyer et al., 2018b) has shown that altering a compass cue in one modality (e.g., magnetic) without a corresponding alteration in compass cues in other modalities (e.g., visual), can introduce cue conflicts (see Figure 6). A nulled field can avoid such conflicts if desired, although cue conflict experiments can be a powerful tool for understanding the interactions of different compass cues. A double-wrapped coil system (Kirschvink, 1992) allows incorporation of an elegant control configuration into the stimulus design. The parallel connection of the coils can be switched to antiparallel connection, supplying the now electronically separated neighboring copper windings of the system with a current of a reversed sign. The resulting local magnetic fields cancel each other out and no magnetic field changes are generated, while the coil system is still operated with electrical current. This results in a true “sham-rotation” of the stimulus which is very useful as a control in behavioral experiments, or to check if, for instance, the coil system itself generates electrical artifacts into nearby electrophysiological equipment. Additionally, the coil system should be carefully grounded.

Our coil system (Figure 5C) – custom built by the workshops of the University of Oldenburg – had outer diameters of 1245 mm (X coils), 1300 mm (Y coils), and 700 mm (Z coils). The coil system is powered by constant-current power supplies, one for each coil axis (Kepco, model BOP 50-2M, 50V, 2A). The current running through the coil systems was controlled via High-Speed USB Carriers (National Instruments USB-9162) and custom-written codes in MATLAB (Mathworks, Natick, MA, United States). A Meda FVM-400 magnetometer, the probe of which is placed at the position of the moth, is used to ensure that the magnetic field is correctly set with the appropriate field parameters for the experiment at hand.

## Experimental Procedures

### Keeping Moths Prior to Experiments

In order to minimize stress, the moths should be stored in a cool, shaded and quiet place, ideally at least one meter above ground (because of ants which might be attracted to the samples). This place should, however, not be totally dark but exposed to the natural light cycle so as not to disturb the moths’ circadian rhythm. We housed our Bogong moths in individual plastic containers which were equipped with cotton buds drenched in honey solution (10%). We recommend using animals for orientation experiments within 3 to 6 days of capture. The cotton buds were replaced with new cotton buds drenched in fresh honey solution every second day. We fed our animals prior to every experiment with fresh honey solution.

### Attaching Tethering Stalks to Moths

To prepare moths for tethering in the flight arena, we adopted a method for attaching tethering stalks to moths that was first established in the lab of Dr. Jason Chapman (University of Exeter, United Kingdom, e.g., Minter et al., 2018). Moths were first calmed by placing them in a freezer for a few minutes and then positioned under a plastic gauze mesh (5 **×** 5 mm mesh holes) secured to a table top on either side of the moth with weights (anything heavy). The thick layer of scales is then removed from the dorsal thoracic plate (the mesoscutum). This can simply be achieved by using a regular small paint brush or a custom-made micro-vacuum equipped with a pipette tip that sucks the scales from the mesoscutum. The micro-vacuum has the advantage of minimizing scale dispersion in the air. In any case, a dust mask is recommended for this procedure. After the scales are removed from the mesoscutum, a ca. 15 mm length of straight tungsten wire (ca. 0.5 mm diameter) is used to make a tethering stalk (this tungsten wire is identical to that used for the encoder axle: *6* in Figure 1). Tungsten wire is an ideal choice as it is non-magnetic and sufficiently stiff. With a pair of needle-nosed pliers, the final 3-5 mm of the tungsten wire is bent into a small loop that is then bent 90° to the rest of the stalk. This loop is glued to the mesoscutum of the moth using Evo-Stik Impact contact adhesive (Evo-Stik United Kingdom), thus furnishing the moth with a vertical tethering stalk. Great care should be taken to avoid damaging/immobilizing the wings or antennae with adhesive, and to position the tungsten stalk perfectly vertically. Once the adhesive is dry, a stalked moth should be kept with fresh food in a plastic container in a cool, shaded and quiet place. For this purpose, we used containers made from UV-transparent Plexiglass. At sunset, prior to the experiments, our stalked moths were placed outside (in individual UV-transmissive Plexiglass containers) on a somewhat elevated position to ensure they could view the setting sun and the celestial rotation for at least 1 h after sunset. Following this, moths were returned to the lab and placed in darkness. Prior to each experiment the moths must be totally dark adapted.

### Insertion of Moths in the Flight Simulator

Even though the apparatus can (with some experience) be operated by one person alone, it is wise to plan for two experimenters to enable a smooth workflow. One person should run the computer, while the other attaches the experimental animals to the simulator prior to each test. Since the experiments should be conducted in more or less absolute darkness, the animals should be handled using a headlamp featuring a dim red LED (invisible to most insects). The experimental moths can easily be extracted from their containers by grasping the tungsten tethering stalk using a pair of regular stainless-steel haemostats. Moths generally fly vigorously when held by the tethering stalk. To tether the moth to the optical encoder, a small length (ca. 10–15 mm) of tightly fitting thin rubber tubing is partially pulled over the free end of the tungsten encoder axle (*6* in Figure 1), i.e., the end that is not connected to the optical encoder. The other free end of the tubing is used to receive the end of the tungsten tethering stalk, which is inserted with the help of the haemostat. This is a very delicate procedure since any permanent bending of the tungsten encoder axle will lead to artifacts in the recorded heading directions – the entire procedure should be practiced in daylight prior to beginning experiments.

The encoder software needs to be calibrated to an external reference direction prior to each experiment. This could be magnetic or geographic North, depending on the experimental design. A light-reflective sticker positioned at North somewhere in the vicinity of the setup turned out to be very helpful for locating this direction. Calibration is achieved by turning the moth on its tether until it is oriented northward and then holding it there until the software encoder direction is zeroed (i.e., a readout of 0° = North). After the system is calibrated, the animal should be given up to a minute to accustom itself to the experimental environment and “settle down” before the recording starts. During this time period the encoder software should be used to check whether the animal can turn in both directions, whether it spirals vigorously in one particular direction (i.e., continuously turns around its tethering axis) or if it stops permanently. If one of these behaviors is displayed it likely indicates a stalking error and the animal should be discarded. In an ideal recording situation, the animal will settle down to a given flight direction after a short while and show a typical behavior which we refer to as “scanning.” This means that the compass needle of the encoder software is hovering over a particular direction on the compass rose, swinging back and forth over a span of about 15°–45°. Using a spirit level, one should occasionally check that the encoder is level since this might influence the flight direction of the animal.

### Experimental Precautions

A necessary first step when using a flight simulator to study the migratory behavior of an insect species is to establish the insect’s natural migratory direction during its migratory season – this can then be used as a control direction for further orientation experiments. While being tested, the animals must experience an unobscured view of the sky and an undisturbed magnetic field. The choice of the experimental location is probably equally as important as the timing of the experiments. “Geographic bottlenecks” along the migratory route, such as mountain passes or valleys, usually concentrate insects during their migration and are often good places for catching sufficient numbers for these experiments.

#### Data Selection

It is reassuring when the recorded natural migratory (control) direction coincides or overlaps with previously established vanishing directions or natural observations, but the experimenter should always be aware of his/her own confirmation bias. The exclusion of a moth from either the experiments or from the analysis should only occur according to pre-determined rules, not according to rules created after the experiments. In our experiments, if a moth performed under ideal outdoor experimental conditions and was still unable to steer a course (irrespective of the direction it chose to fly), and its resulting trajectory had an *r* value less than 0.2, this moth was excluded from the analysis. However, to compare indoor orientation experiments under different stimulus conditions, no lower threshold for the *r* value should be set because disorientation might be a valid outcome of the experiment due (say) to the presentation of a deliberate cue conflict between two or more of the applied stimuli. Thus, in this case, a low *r* value might be an expected outcome and filtering out this particular moth might mask the effect of a natural behavior.

It sometimes happens that even a seemingly well-oriented moth stops performing flight behavior before the previously determined experimental time is over. If this occurred, we usually tried to kick-start the animal by gently bumping the arena. If a moth stopped 4 times during an experiment, we aborted it. In particularly unsettled weather conditions, such as a looming thunderstorm, we found that the moths were not eager to perform in the arena and frequently stopped flying (and this occurred both during indoor and outdoor experiments).

#### Moon Phase and Weather

Even if the moon’s disc is not directly visible to the animal, the moonlight entering an outdoor arena can introduce an intensity gradient on the wall of the arena situated opposite to the physical direction of the moon’s disc. This uneven illumination of the arena wall could provide unwanted (and confounding) orientation cues for the flying moth. It is possible to shade the arena from moonlight using a flat piece of plywood or commercially available sunshades (e.g., a beach umbrella), but this might block a considerable part of the sky which in turn could interfere with the experimental design. Moreover, any top-heavy structure with a large surface is very vulnerable to be blown over by the wind. When choosing a suitable time window for outdoor experiments, the current moon phase, prevailing winds, predicted precipitation and temperature are important factors to account for and to monitor. If possible, the dew point spread should also be monitored during an experimental night as we found that moths began to behave erratically in the arena if there was too much moisture in the air (Dreyer et al., 2018a).

#### Putative Artifacts

Since many animal species are attracted to landmarks in behavioral experiments, great care must be taken to avoid unwanted landmarks, such as treetops in outdoor experiments, being visible from the inside of the arena. The easiest way to check for this is to set up the arena at the same height above ground as it is intended to be located during an experiment and to visually confirm that no outside landmarks are visible from the inside of the arena by sticking one’s head through the bottom of the arena.

Any stray light generated by the equipment must be avoided since this too could provide an unwanted orientation cue that could affect the heading direction of a tested moth. This includes the screen of the recording computer and the reflection of the screen light on the face of the experimenter. The computer screen should be set to the lowest possible intensity setting and covered with a thin sheet of red plastic filter to block out most wavelengths visible to insects (such filter sheets can be obtained from Lee Filters). The recommended use of red LEDs during the experiments has already been mentioned. A red-light regime will make it very difficult to read or identify handwritten notes or markings which were made using a pen or marker with red ink. To check if the walls of the arena are impermeable to artificial stray light from the outside, it is very helpful to put a very bright light source on the inside of the arena and to look for stray light shining through cracks and irregularities from the outside.

### Experimental Design for Orientation Experiments

In previous orientation experiments in which a migratory behavior was convincingly demonstrated to be driven by the animal’s orientation relative to a particular compass cue, the animal’s orientation could be altered by changing the position or orientation of that cue (e.g., Kramer, 1950; Wiltschko and Wiltschko, 1972; Emlen, 1975; Lohmann, 1991).

**FIGURE 7:**
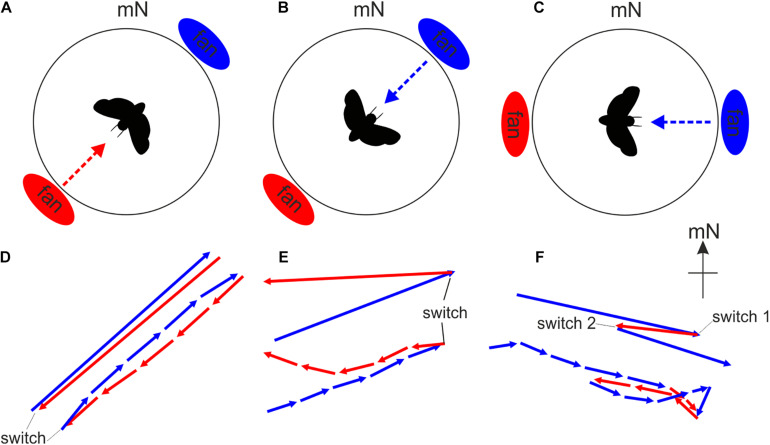
The modified Mouritsen-Frost flight simulator can be used to monitor changes in flight behavior in response to changes in putative orientation cues. Since wind speed and direction influence the migratory behavior of moths (e.g., Chapman et al., 2008b), we exposed migratory Bogong moths to very weak air streams (6 kph) from two different directions relative to magnetic North while they performed flight behavior in our arena. The air streams were generated by two small fans. **(A,D)** The AB stimulation sequence. The fan located in the southwest was activated (*red dashed arrow*) and the animal flew for 5 min (condition A). We found that moths fly roughly toward the direction of the wind stimulus (i.e., into the wind), as seen by the *red flight trajectory vector* shown in **D**. The *upper* vectors in panels **D–F** indicate the entire average 5 min flight while the lower vector sequence indicates the flight behavior within each successive 1-min bin. The length of each vector indicates the “directedness” of the flight, that is, the fidelity with which the moth kept to the same flight direction. Directly following condition A, the fan located in the northeast was switched on and the animal flew for another 5 min (condition B), again into the wind as seen by the *blue flight trajectory vector* shown in **D**. **(B,E)** The BA stimulation sequence. The same procedure as in **A,D** but with the wind stimulus presented in the reverse sequence. **(C,F)** The ABA stimulation sequence. Here the fans were rotated by 45° to form an east-west axis. The fan located in the east was activated first (*blue dashed arrow*) and the animal flew for 5 min (condition A). Then the fan located in the west was activated for 5 min (condition B). Finally condition A (east fan activated for 5 min) was repeated.

One classic approach is the ABA stimulus configuration (Figure 7). In an orientation experiment, this entails an animal being asked to perform migratory orientation behavior relative to a particular cue (condition A). In our illustrated example, this cue is a weak wind stream provided by a small fan mounted into the arena wall (Figure 7) – Bogong moths respond to this wind stream by flying somewhat into it. In a second experimental condition, the spatial orientation of this cue is altered (e.g., the position of the fan is shifted by 180°: condition B). This experimental sequence is referred to as an AB sequence (Figure 7A), and this can be used to determine whether the moth truly responds to the cue (which in this case means that the moth should turn roughly 180° from A to B, as indeed it does: Figure 7D). Reversing the order of the experimental conditions (i.e., a BA sequence) can be used to confirm the orientation response (Figures 7B,E). An ABA stimulus configuration (Figures 7C,E) is a classic configuration which seeks to confirm that the behavior observed initially can be restored and is thus truly related to the change in spatial orientation of the compass cue. The results of a classic ABA experiment become even more convincing when the ABA sequence is exchanged for a BAB sequence in 50% of the experiments without a noticeable change in the conclusions that can be drawn from the results, and if control experiments (e.g., AAA, BBB or a control condition featuring no relevant orientation-related information, CCC), alternating with the actual experiments, lack the previously observed changes in the behavior of the animal.

In the case of Bogong moths, we discovered that most of the animals are extremely sensitive to the presence of unintentionally presented visual landmarks (an irregularity in the felt on the wall of the arena, a scratch in the lid holding the encoder, etc.). This becomes problematic if tested under condition B since any compass cue which is systematically changed in condition B is now set in conflict with the previously learned spatial relationship of this cue with the unintentionally presented landmark, which can confuse the moth. In our earliest experiments we discovered that this led to clearly less oriented flight behavior during condition B. We took advantage of this “sensitivity” toward landmarks in later experiments by employing obvious and intentional visual landmarks within the arena. This allowed us to design cue conflict experiments which demonstrated that Bogong moths are able to sense the Earth’s magnetic field and that they learn the relationship between this magnetic field and visual landmarks to steer migratory flight (Dreyer et al., 2018b).

## Analysis of Orientation Data

The results of the cue conflict experiment on Bogong moths mentioned above (Figure 6B) provide a good introduction to the methods we have used to analyse data generated in the flight arena (Dreyer et al., 2018b). In these experiments, 42 moths were each allowed to fly for 5 min while exposed to a conspicuous visual cue (a triangular black “mountain” above a lower black “horizon” within the flight simulator arena, and a black stripe on a rotatable circular UV-transmissive diffuser above the moth) and an earth-strength magnetic field (Figure 6B). These two cues – visual and magnetic – were either turned together while maintaining their learned correlated arrangement (Figure 6B), or one cue was turned without the other to create a cue conflict (Figure 6B). Whenever the cue correlation was maintained, the population of moths remained oriented, but when a cue conflict was introduced, they became disoriented, implying that both visual and magnetic cues are used for steering migratory flight (Dreyer et al., 2018b).

These results were derived by analyzing the 42 moths as a single population. For each of these moths, our recording system, as previously mentioned, allows us to record the virtual trajectory of each moth by sampling its orientation choices as angles relative to gN at a frequency of 5 Hz (Figures 4A,B). Based on these angles, custom-written software and the MATLAB Circular Statistics Toolbox (Berens, 2009) were used to calculate an average vector representing the moth’s trajectory, the direction and length of which reveal the mean orientation angle and directedness of the moth, respectively – these are the gray vectors in the circular data plots for Red underwing moths shown in Figure 4C (14 vectors for the 14 moths flown) and for Bogong moths shown in Figure 7 (42 vectors for the 42 moths flown). The length of the vector is reflected in its *r* value (a unitless value between 0 and 1) – the longer the vector, the greater the *r* value and the more consistently the moth flew in its chosen direction.

**FIGURE 8:**
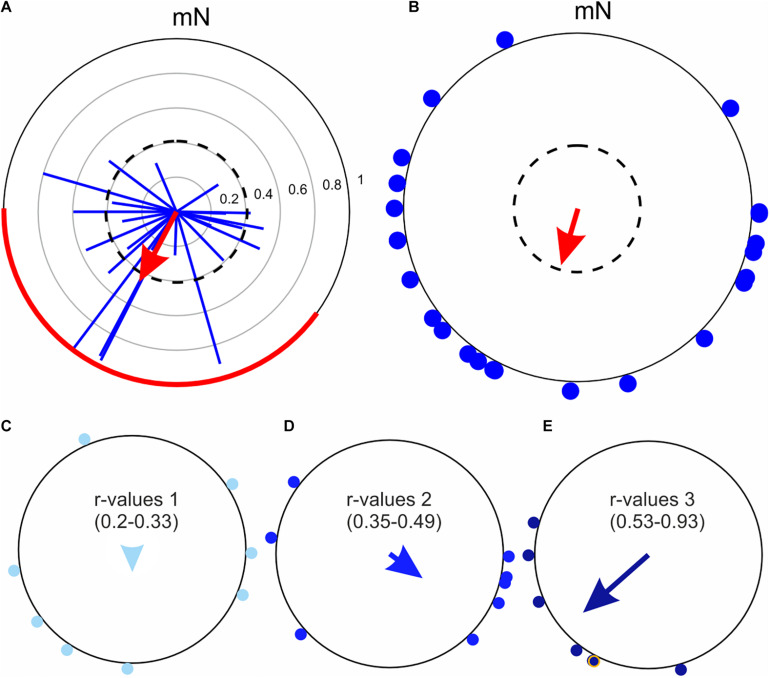
A comparison of the non-parametric Moore’s modified Rayleigh test (MMRT) and the classical Rayleigh test, using the flight trajectories of 23 Dark sword-grass moths (*Agrotis ipsilon*) recorded at Col de Coux in Switzerland. **(A)** Flight trajectories analyzed using the MMRT. The individual flight trajectory vectors of each moth are shown as *blue vectors* and the average heading vector of the population (sample) derived from the test is shown as the *red arrow*. The *dashed circle* indicates the required R* value for statistical significance (*p* < 0.05) and the *red line* on the outer circle marks the 95% confidence interval. The *thin gray circles* indicate the *r* value (in steps of 0.2), which are applicable to the flight trajectory vectors of individual moths (*blue vectors*). **(B)** Same data as in A, but now evaluated using the classic Rayleigh test. The mean flight directions of each moth are shown as *blue dots* around the periphery of the circle. According to the classic Rayleigh test, which does not weight the orientation choices according to their *r* value (as does the Moore’s modified Rayleigh test, A), the population is not significantly oriented. The *dashed circle* indicates the required α-level for statistical significance (*p* < 0.05). Note that the length of the *red arrow* in B encodes the *r* value, not the R* value. **(C–E)** The mean flight directions of individual moths (from panel **B**) were ranked according to the lengths (*r* values) of their underlying flight trajectory vectors (from panel **A**) and accordingly assigned to three bins: *r* values 0.20–0.33 (**C**, *n* = 8), *r* values 0.35–0.49 (**D**, *n* = 8), and *r* values 0.53-0.93 (**E**, *n* = 7). The mean vectors for each of the three sub-populations were computed using only the mean flight directions of the moths (*arrows* in each plot). Moths with flight trajectory vectors having larger *r* values **(C)** tend to cluster more tightly around a single orientation direction (leading to a longer mean sub-population vector).

Once we have determined the average vectors for each of the 42 moths, we can investigate the behavior of the moths as a single population. To do this, we apply a non-parametric Moore’s modified Rayleigh test (MMRT: Moore, 1980; Zar, 1999), calculated using the circular statistics software Oriana (KCS, Pentraeth, United Kingdom). The MMRT ranks the vectors according to their length (i.e., *r* value) and weights them according to these ranks, meaning that not only the mean direction of a moth’s vector, but also its directedness (length), impacts the ultimate outcome of the test – the generation of an average heading vector for the population as a whole (for a detailed description of the statistics involved, see Dreyer et al., 2018b). This average population vector – shown as the red vector in each of the circular data plots of Figure 6 – has a length that indicates the likelihood that the population is heading in the specific direction indicated by the vector. This length is represented by the vector’s *R*^∗^ value (see Figures 6B, 8 for details). The greater the *R*^∗^ value, the more directed is the population it represents.

A significant advantage of knowing the entire virtual flight trajectory of each moth is that one has access to much more information. In addition to knowing the moth’s average heading direction (trajectory vector direction), one also knows how well directed the moth was during its flight (trajectory vector length).

When a trajectory exists, the advantage of the MMRT over the regular Rayleigh test (Batschelet, 1981) becomes apparent (Figure 8). An MMRT analysis of the flight trajectory vectors of 23 Dark sword-grass moths (*Agrotis ipsilon*), recorded at Col de Coux in Switzerland (Figure 8A), is compared to a classic Rayleigh analysis of their heading directions alone (Figure 8B). A significant average heading vector for the population only appeared after accounting for the directedness of the 23 moths by using the MMRT test (red vector in Figure 8A, *p* < 0.05). A classic Rayleigh test (ignoring directedness) on the same data indicates that the moths were instead disoriented (red vector in Figure 8B, p = ns). The reason for the difference lies in the fact that for this data set (and many other flight-simulator data sets we have observed), more directed moths (i.e., moths with flight trajectory vectors having larger *r* values) tend to cluster more tightly around a single orientation direction (leading to a longer average subpopulation vector, Figure 8E), whereas less directed moths tend to have average heading directions that are somewhat more random (Figures 8C,D). Since the MMRT gives greater weight to more directed individuals, this test finds a significant orientation direction for this population of Dark sword-grass moths (Figure 8A).

Both the Rayleigh test and the MMRT operate on the null hypothesis that the orientation choices are uniformly distributed around a circle (Batschelet, 1981). However, in the case of a rejection of the null hypothesis, both tests assume a circular normal distribution, meaning that the distribution of data is unimodal (i.e., possesses a single cluster of orientation choices). If a bimodal distribution of orientation choices is to be expected, the mean orientation angle of each individual animal can first be transformed by doubling this mean angle (if the resulting angle is greater than 360°, one must subtract 360° from this result). Once this is done, one is free to test the modified dataset using the MMRT or Rayleigh test.

Finally, in order to determine whether the distributions of orientation choices made by two different populations (or samples) are significantly different, we employ the non-parametric Mardia-Watson-Wheeler uniform-scores test (Batschelet, 1981), calculated using Oriana. This proved useful in our studies of Bogong moths, where tested populations of autumn and spring migrants were expected to migrate in significantly different directions (and indeed did so: Dreyer et al., 2018b). The Mardia-Watson-Wheeler test can also be used for determining whether populations of two different species possess the same or different migratory headings (Dreyer et al., 2018a).

## Conclusion

The Mouritsen-Frost flight simulator was initially designed to record the orientation choices of diurnal insects during their migration (Mouritsen and Frost, 2002). Relative to their “natural orientation behavior,” a subpopulation of tethered flying insects can then be tested under conditions in which the spatial orientation of a putative compass cue (or several cues) is altered, with the goal of determining whether the insects compensate for this alteration. Apart from this obvious application, one can also use the flight simulator to investigate the influence of external “disturbance factors,” such as an artificial light stimulus of certain intensity, polarization, and/or wavelength, on the flight performance of insects. Such methods could for instance also be used to investigate the influence of other stressors, such as light pollution on insect migration, or to investigate the influence of various types and concentrations of pesticides on the migratory flight capacities of different insect species.

A technically more advanced application is to integrate the flight simulator within an electrophysiology rig, as is being successfully done to monitor the neuronal activity of brain areas involved in navigation while an insect is tethered within the arena (Beetz et al., in preparation). In these experiments, an extracellular tetrode array (containing typically 4-5 electrodes) can be inserted into the brain while the insect performs flight behavior in the arena under controlled stimulation conditions. The tetrode enables the experimenter to pick up neuronal responses from several neurons at once (typically 2–5 per electrode), increasing the chances of encountering neurons involved in the processing of navigational information. Changes in the firing rates of recorded neurons could subsequently be correlated to changes in the spatial orientations of external sensory stimuli and to changes in flight direction that these may induce. Such methods would constitute powerful tools for dissecting the function of neural networks responsible for processing and acting on sensory information encountered during migration and navigation.

## Data Availability Statement

The raw data supporting the conclusions of this article will be made available by the authors upon request, without undue reservation.

## Author Contributions

AL and DD conducted the flight-simulator experiments in Switzerland and analyzed the data. MM assisted with experiments and fieldwork in Switzerland. HM and DD recorded the preliminary dataset presented in Figure 6. DD, EW, HM, and BF provided their experiences gained by running different experimental designs in the flight simulators over the course of many years. DD and EW made the figures and wrote the initial version of the manuscript. All authors made significant contributions to the final version.

## Conflict of Interest

The authors declare that the research was conducted in the absence of any commercial or financial relationships that could be construed as a potential conflict of interest.
